# Leishmaniasis, Autoimmune Rheumatic Disease, and Anti–Tumor Necrosis Factor Therapy, Europe

**DOI:** 10.3201/eid1506.090101

**Published:** 2009-06

**Authors:** Ioannis D. Xynos, Maria G. Tektonidou, Dimitrios Pikazis, Nikolaos V. Sipsas

**Affiliations:** National and Kapodistrian University of Athens, Athens, Greece (I.D. Xynos, D. Pikazis, N.V. Sipsas); Euroclinic Hospital of Athens, Athens (M.G. Tektonidou)

**Keywords:** Anti-TNF, tumor necrosis factor, leishmaniasis, rheumatic disease, autoimmune diseases, parasites, Europe, dispatch

## Abstract

We report 2 cases of leishmaniasis in patients with autoimmune rheumatic diseases in Greece. To assess trends in leishmaniasis reporting in this patient population, we searched the literature for similar reports from Europe. Reports increased during 2004–2008, especially for patients treated with anti–tumor necrosis factor agents.

We report 2 new cases of leishmaniasis involving patients with autoimmune rheumatic diseases who received anti–tumor necrosis factor (anti-TNF) agents. We also reviewed all similar cases from Europe reported in the literature, and we discuss the implications of leishmaniasis in the setting of anti-TNF therapy, which is associated with increased risk for opportunistic infections (*1*).

## The Study

Patient 1, a 55-year-old man who had received a diagnosis of ankylosing spondylitis 7 years previously, was admitted to Laikon Hosptal, Athens, Greece, in May 2005 for evaluation of encrusted vesicular lesions on the face. The lesions were painless but mildly pruritic. The patient had been receiving nonsteroidal antiinflammatory agents until 12 months before admission, when his medications were changed to infliximab (3 mg/kg) plus methotrexate (10 mg weekly) because of his deteriorating clinical condition. He was living in a leishmaniasis-endemic area in Athens, had no pets in his house, and had no history of recent travel abroad. The central scale was removed from one of the lesions, and scrapings from the base of the lesion were stained with Giemsa stain, which showed intracellular amastigotes with peripheral nuclei and rod-shaped kinetoplasts. Results of indirect immunoflorescent antibody (IFA) testing were positive for *Leishmania* parasites (titer 6,400). Infliximab and methotrexate therapy was discontinued, and treatment with liposomal amphotericin B was started at a dose of 3 mg/kg, for days 1 to 5, and 2 additional doses (3 mg/kg) on days 14 and 21. Eighteen months later, treatment with etanercept was begun due to the patient’s severe spondyloarthritis; 2 years after the new anti-TNF treatment, he is well, with no signs or symptoms of leishmaniasis.

Patient 2, a 71-year-old woman who had giant cell arteritis, was admitted to the Euroclinic Hospital, Athens, in May 2005 with a high fever and fatigue. The patient had been treated with infliximab (0.25 mg/kg) and variable doses of methylprednisolone for the previous 2 years. Methotrexate (10 mg/week) was added 1 year before admission. She was also living in an Athens suburb, which is leishmaniasis-endemic, and had 4 dogs. Laboratory tests showed a high level of C-reactive protein (163 mg/L, reference range 0–6 mg/L), high erythrocyte sedimentation rate (77 mm/h), pancytopenia (hemoglobin level 12.5 g/dL, leukocyte count 3,300/mm^3^, platelet count 122,000/ mm^3^), and diffuse hyperglobulinemia. The examination of Giemsa-stained smears from bone marrow aspirate demonstrated abundant *Leishmania* parasites, and IFA was marginally positive for *Leishmania* antibodies (titer 400). PCR was positive for the detection of the *Leishmania* genome in peripheral blood. Infliximab and methotrexate treatment was discontinued, and treatment with intravenous liposomal amphotericin B was started at a dose of 3 mg/kg for 5 days. Two days later, the fever subsided, and within the next few days, the patient recovered from pancytopenia, while the inflammatory markers showed a gradual decrease. She received 2 additional doses of liposomal amphotericin B (3 mg/kg) on days 7 and 14, and by that time, she exhibited no signs or symptoms of visceral leishmaniasis.

We then searched Medline, EMBASE, and Current Contents databases for all reports on leishmaniasis in Europe and the Mediterranean area among patients with autoimmune rheumatic diseases, which are often treated with anti-TNF agents. In our search strategy, we used medical subject heading terms and text words, including rheumatoid arthritis, juvenile rheumatoid arthritis, Still’s disease, seronegative arthritis, psoriatic arthritis, Behçet’s disease, ankylosing spondylitis, reactive arthritis, vasculitis, giant cell arteritis, Wegener’s granulomatosis (ANCA [anti-neutrophil cytoplasmic antibody]–associated vasculitis), panarteritis nodosa, leishmaniasis, *Leishmania*, and anti-TNF. We searched the reference list of each resulting report for additional publications. We used no language or time restrictions.

All retrieved articles were case reports. We found 13 additional cases of leishmaniasis in patients with autoimmune rheumatic diseases (*2*–*14*), all published after the introduction of anti-TNF agents in 1998 (Table). All 15 patients (including our 2 patients) were treated in the past or at the time of the diagnosis of leishmaniasis with >1 standard immunosuppressive agents, including corticosteroids (11/14 [78.5%]) patients for whom treatment details were reported), methotrexate (9/14 [64.3%]), cyclosporine (3/14 [21.4%]), cyclophosphamide (3/14 [21.4%]), azathioprine (2/14 [14.3]), and chlorambucil (1/14 [7.1%]). Seven (46.6%) patients received an anti-TNF agent along with standard immunosuppressive agents. Two of the 15 reported patients had been treated with a recombinant interleukin–1 receptor antagonist (anakinra; Amgen Inc., Thousand Oaks, CA, USA) (*5*,*13*). In most of the patients, visceral leishmaniasis developed (13 patients, 86.6%), while cutaneous leishmaniasis developed in 2 patients (1 was receiving an anti-TNF agent). All patients were living in leishmaniasis-endemic areas of Europe (Figure).

**Table:** Detailed characteristics of 15 patients with autoimmune rheumatic disorders in whom leishmaniasis developed, Europe*

Patient no.	Country	Age, y/sex	Disease	Anti-TNF treatment			Other immunosuppressive treatments		Form of *Leishmania* infection	Ref.
				Agent	Duration, mo		Agent(s)	Duration, mo		
1	France	66/M	ANCA-associated vasculitis	NA	NA		Cyclophosphamide, methotrexate, corticosteroids	120	Visceral	(*7*)
2	Israel	56/M	Rheumatoid arthritis	NA	NA		Methotrexate, corticosteroids	120	Cutaneous	(*8*)
3	Italy	35/M	Behçet disease	NA	NA		Chlorambucil, corticosteroids	36	Visceral	(*9*)
4	Spain	50/M	Rheumatoid arthritis	NA	NA		Methotrexate, corticosteroids	120	Visceral	(10)
5	Italy	60/M	Polyarteritis nodosa	NA	NA		Cyclophosphamide, corticosteroids	2	Visceral	(*11*)
6	Spain	55/M	Psoriatic arthritis	Infliximab	9		No details given	300	Visceral	(*2*)
7	Italy	76/M	ANCA-associated vasculitis	NA	NA		Cyclophosphamide, corticosteroids	36	Visceral	(*12*)
8	France	53/F	Rheumatoid arthritis	Infliximab	12		Azathioprine, corticosteroids	12	Visceral	(*3*)
9	Italy	69/F	Rheumatoid arthritis	Adalimumab	25		Methotrexate, corticosteroids	360	Visceral	(*4*)
10	Greece	60/F	Rheumatoid arthritis	Etanercept	18†		Cyclosporine, corticosteroids, anakinra	96	Visceral	(*5*)
11	France	9/F	Juvenile rheumatoid arthritis	NA	NA		Cyclosporine, methotrexate, corticosteroids, anakinra	60	Visceral	(*13*)
12	Greece	45/M	Psoriatic arthritis	Infliximab	60		Methotrexate, corticosteroids	60	Visceral	(*6*)
13	Greece	65/F	Rheumatoid arthritis	NA	NA		Methotrexate	96	Visceral	(*14*)
14	Greece	71/F	Giant cell arteritis	Infliximab	24		Methotrexate, corticosteroids	24	Visceral	This study
15	Greece	55/M	Ankylosing spondylitis	Infliximab	12		Methotrexate	12	Cutaneous	This study

*TNF, tumor necrosis factor; Ref., reference; ANCA, anti-neutrophil cytoplasmic antibody; NA, not applicable. †All biologic treatments had been terminated 6 mo before leishmaniasis occurred.

**Figure:**
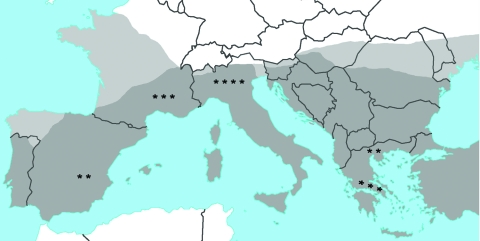
Reported cases of leishmaniasis in patients with autoimmune rheumatic diseases in Europe, indicated by stars (1 case from Israel not shown). Dark gray shading, distribution of leishmaniasis; light gray shading, distribution of leishmaniasis vector sandfly. Source: World Health Organization, 2004 (www.who.int/tdr/svc/publications/tdr-research-publications/swg-report-leishmaniasis).

The anti-TNF agents were introduced into clinical practice in 1998, and the first case of leishmaniasis associated with anti-TNF blockade occurred in 2001 (*4*). During the 6-year period (1998–2003), a total of 6 reports were made of leishmaniasis in patients with rheumatic diseases; 1 (16.6%) occurred in a patient treated with an anti-TNF agent. During the ensuing 5 years (2004–2008), 9 cases of leishmaniasis were reported, 6 (66.6%) in patients receiving anti-TNF agents.

The median duration of previous immunosuppressive therapy, before the diagnosis of leishmaniasis, was 60 months (range 2–360 months) for all 15 patients (Τable). For the 7 patients who received anti-TNF agents, the median duration of anti-TNF treatment was 18 months (range 9–60 months). Six of these 7 patients were receiving anti-TNF agents when symptoms and signs of leishmaniasis occurred. In 1 patient (*5*), biologic treatments had been discontinued 6 months before the diagnosis of leishmaniasis (Table). Only 1 patient had been tested for antibodies against *Leishmania* spp. before immunosuppressive therapy was begun, and the results were negative (*6*). Therefore, this is the only case with compelling evidence that leishmaniasis was a primary infection and not reactivation of a latent infection.

## Conclusions

Our data suggest that the introduction of TNF blockade into the clinical practice is associated with increasing reports of leishmaniasis in patients with autoimmune rheumatic diseases who live in leishmaniasis-endemic areas of Europe. Notably, in most reported cases, patients had not received anti-TNF agents but other immunosuppressants. However, all cases of leishmaniasis in patients with autoimmune rheumatic diseases were reported after 1998, the year of introduction of anti-TNF agents, and most (9/15) of the reported leishmaniasis cases occurred during the past 5 years (2004–2008), mainly among patients receiving anti-TNF agents (6 of the 9 patients with leishmaniasis; 66.6%). This increase coincides with the increasing use of anti-TNF agents during the same period, as prescription practice started changing toward treating patients with lower disease activity (*15*). Another indirect piece of evidence that TNF blockade may increase the risk for leishmaniasis is that the median duration of previous anti-TNF treatment before the diagnosis of leishmaniasis was significantly shorter than the median duration of immunosuppressive therapy for all 15 patients (18 vs. 60 months).

Our report has limitations. It is unclear for all cases (with 1 exception) presented in this article whether leishmaniasis was primary infection or reactivation of latent disease. We cannot also exclude the possibility that the concomitant, long-term use of other immunosuppressants, and not the anti-TNF agents per se, played a crucial role in the development of leishmaniasis. Different prescribing patterns of anti-TNF agents might influence the number of cases reported from each disease-endemic European country. However, the small number of reported cases and the lack of data on differences in the anti-TNF prescribing policies do not allow any conclusions to be reached. Finally, due to underreporting, the reported cases may underestimate the real incidence of leishmaniasis among patients with autoimmune rheumatic diseases.

Prospective studies to estimate the incidence of the disease, the impact of risk factors and the need for serologic screening for leishmaniasis before initiation of anti-TNF agents or any other immunosuppressive treatment are clearly needed. This is particularly important since currently only a few patients with autoimmune rheumatic diseases receive anti-TNF agents (*15*). Therefore, the use of anti-TNF treatment is likely going to increase, possibly causing a parallel increase in opportunistic infections such as leishmaniasis.
